# Killing Traps and Snares in North America: The Need for Stricter Checking Time Periods

**DOI:** 10.3390/ani9080570

**Published:** 2019-08-17

**Authors:** Gilbert Proulx, Dwight Rodtka

**Affiliations:** 1 Alpha Wildlife Research & Management Ltd., 229 Lilac Terrace, Sherwood Park, Alberta, AB T8H 1W3, Canada; 2 Retired Problem Wildlife Specialist, Alberta Agriculture, Box 1366, Rocky Mountain House, Alberta, AB T4T 1B1, Canada

**Keywords:** AIHTS, killing traps, killing snares, wildlife welfare, trap check times, trapline management, international humane trapping standards

## Abstract

**Simple Summary:**

In this review, we make the point that current checking times for killing traps and snares are inadequate or nonexistent in most North American jurisdictions. We use Conibear 120 rotating-jaw traps and killing neck snares as examples of trapping devices that may fail to consistently and humanely kill furbearers. Because these killing devices are not powerful enough for the target species, the trigger systems do not properly position the animals in traps, or trappers are inexperienced and improperly set traps or snares, these killing devices become restraining devices, and animals suffer long and painful deaths. Because trappers use a variety of trigger configurations and trap sets, all killing devices, even those certified by trapper organizations or governments, should be monitored at least once every 24 h on traplines, but preferably every 12 h, because one cannot know a priori whether traps will strike animals in appropriate locations for a quick kill. However, when using trapping devices such as killing neck snares that are legal and allowed by government agencies despite being inhumane, trappers should check them every 12 h. When traplines are situated near urban areas, e.g., within 10 km, checks should be done every 12 h to release pets and non-target animals.

**Abstract:**

In this review, we make the point that current checking times for killing traps and snares are inadequate or nonexistent in most North American jurisdictions. We use Conibear 120 rotating-jaw traps and killing neck snares as examples of trapping devices that may fail to consistently and humanely kill furbearers. Because these killing devices are not powerful enough for the target species, the trigger systems do not properly position the animals in traps, or trappers are inexperienced and improperly set traps or snares, these killing devices become restraining devices, and animals suffer long and painful deaths. Because trappers use a variety of trigger configurations and trap sets, all killing devices, even those certified by trapper organizations or governments, should be monitored at least once every 24 h on traplines, but preferably every 12 h, because one cannot know a priori whether traps will strike animals in appropriate locations for a quick kill. However, when using trapping devices such as killing neck snares that are legal and allowed by government agencies despite being inhumane, trappers should check them every 12 h. When traplines are situated near urban areas, e.g., within 10 km, checks should be done every 12 h to release pets and non-target animals.

## 1. Introduction

Since 1995, organized efforts to reform animal trapping were aimed primarily at reducing cruelty to animals, particularly by outlawing the steel-jawed leghold trap [1]. In the last 40 years, however, there has been a growing societal concern regarding the issue of “humaneness” in wildlife trapping [1]. Trap research programs have been conducted in North America to identify or develop humane killing traps, i.e., traps that quickly render target animals unconscious and minimize pain and suffering [2]. Researchers also recommended that humane trapping standards be adopted to ensure that animals are either live-captured with minimal distress and trauma, or killed as quickly as possible, insofar as the state of the science or the art will allow [1].

According to the Agreement on International Humane Trapping Standards (AIHTS) [3], and the Agreed Minute between the European Community and the United States of America on humane trapping standards [4], Canada and the USA agreed to promote the use and application of traps and trapping methods for the humane treatment of animals. In their respective agreements, they indicated that, although welfare can vary widely, the term “humane” is used only for those trapping methods where the welfare of the animals is maintained at a sufficient level. They also acknowledged that in certain situations with killing traps, “*there will be a short period of time during which the level of welfare may be poor*”. Both agreements set the time limits to unconsciousness to 45 s for *Mustela erminea*, 120 s for *Martes americana*, *Martes zibellina* and *Martes martes*, and 300 s for all other species in 80% of 12 tested animals [3,4]. In 20% (2 animals) of tests, poor welfare conditions may exceed these limits, likely by a few minutes only. In the context of this paper, poor animal welfare would relate to animals in pain while conscious, deprivation of water and food, increased heart rates and raised levels of corticosteroids (‘stress hormones’), and incapability of the animals to cope with pain or discomfort [5,6].

Trap testing in semi-natural environmental conditions has shown that, with some killing traps and snares, animals would not have lost consciousness within the AIHTS’ time limits and could have stayed alive for long periods of time if the researchers had not anesthetized them [7,8]. Work on traplines also showed that killing traps and snares were not always performing as expected, and ≥30% of animals captured in legal traps in Canada and the USA were struck in non-lethal regions and lost consciousness many minutes past the acceptable time limit, or were still alive for hours after capture [9,10]. Nevertheless, according to Dave Kay (2019, Fish and Wildlife Policy Branch, Alberta Environment and Parks, personal communication with Rodtka), check times are irrelevant for killing traps and snares because the animals should be dead at time of visit. Because trappers use a variety of trigger configurations and trap sets, one cannot be sure that animals will be struck in lethal regions. Without knowing a priori whether traps have struck animals in appropriate locations for a quick kill, assuming that traps worked as advertised and humanely killed all captures may lead to long and painful suffering, and poor levels of animal welfare [2,10].

In this review, we make the point that current checking times for killing traps and snares are inadequate or nonexistent in North American jurisdictions. Also, on the basis of published records of animals that were alive and conscious for long periods of time in killing traps and snares, we propose changes to current trapping practices to include stricter time limits in regulations for checking killing traps and snares.

## 2. Checking Times of Kill Traps and Snares in North American Jurisdictions

We consulted the trapping regulations of Canadian Provinces and Territories, and of American States, to determine checking times for killing traps and killing neck (body) snares (Appendix A). These regulations are subject to revision from year to year. At time of writing, in Canada, there are no legal requirements to check killing traps and snares in most Provinces and Territories (Appendix A). In nearly 35% of American jurisdictions, checking times for killing traps and snares exceed 24 h. In approximately 55% of American States, checking times for submersed killing devices exceed 36 h (Appendix A). In both countries, checking times for killing traps and snares often are longer than those of restraining traps which usually are 24 h (Appendix A).

## 3. Animals Restrained in Killing Traps and Snares: Three Case Studies

Killing traps and snares do not always kill animals quickly. Animals that are being restrained in such trapping devices may take hours or even days to die depending on the trapping device, the capture location, the physical condition of the animals, and the environmental conditions. In the following, we review examples of traps and snares that have been found to be ineffective to consistently kill animals humanely, even though they are either “AIHTS-certified” as being humane for some species in Canada or considered in Best Management Practices (BMPs) in the United States. BMPs are educational guides designed to address animal welfare and increase trappers’ efficiency and selectivity.

### 3.1. The Conibear 120 Rotating-Jaw (Bodygrip) Trap Model to Kill Marten

According to AIHTS [3], a killing trap would meet the standards if 80% of 12 tested animals are unconscious and insensible within a pre-determined time limit (e.g., 2 min for small mammals like martens), and remain in this state until death. This means that, on the basis of the normal approximation to the binomial distribution (one-tailed test) [11], a humane trap would, with 95% confidence, render ≥58% of captured animals irreversibly unconscious within the prescribed time limit.

**Figure 1:**
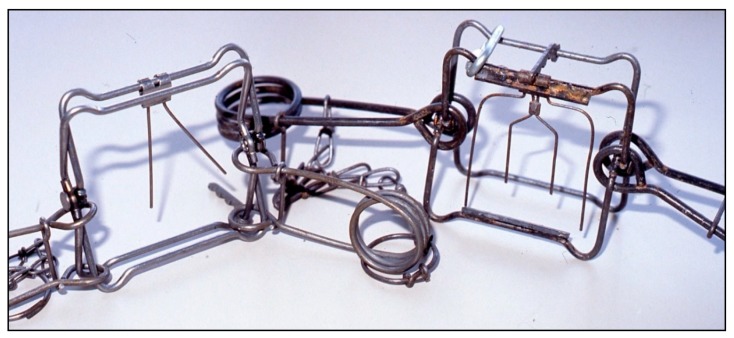
Examples of the Conibear 120 trap with a two-prong trigger (left) and the C120 Magnum trap with a pitchfork trigger (right). Note the larger springs and clamping bars welded to the striking jaws of the C120 Magnum (Photograph: Gilbert Proulx^©^).

The Conibear 120 trap (Woodstream Corp., Lititz, PA, USA; Figure 1) is the most commonly used trap to harvest American martens (*Martes americana*) in North America [7]. It is not certified as humane for marten in Canada [12], but is part of the USA BMPs [13].

The impact and clamping energies of this trap are lower than the kill threshold standards of the Canadian General Standards Board (CGSB) for American martens [14] where animals must be rendered irreversibly unconscious in ≤3 min. Mechanical evaluations showed that the Conibear 120 trap does not have the potential to render animals unconscious in ≤3 min [15] and thus to meet AIHTS’ 2-min time limit. This was further demonstrated in tests with wild animals in simulated natural environments [7] where 2 out of 6 tested animals did not lose consciousness within 5 min (the time limit was 3 min but the research protocol allowed researchers to prolong it to 5 min to learn more about traps). This result suggests that, based on the normal approximation to the binomial distribution (one-tailed) [11], the Conibear 120 trap would then be expected to humanely kill (by rendering animals unconscious in ≤3 min as per CGSB), with 95% confidence, >20% of all captured martens of a true population. The poor performance of the Conibear 120 trap to humanely kill martens was further determined on working traplines [9]. At least 4 out of 13 martens captured in Conibear 120 traps were struck in non-lethal regions that would not result in a loss of consciousness in ≤3 min. Thus, on the basis of a one-tailed binomial test, the trap would, with 95% confidence, render <40% of captured martens unconscious in ≤3 min.

The Conibear 120 trap is still available on the market; it can be purchased at trapper supply stores and through the internet, and it is commonly encountered on traplines (Proulx, unpublished observations), simply because traplines are not being monitored by Conservation Officers, and standards are not being enforced. The inability of the Conibear 120 trap to humanely kill American martens led to the development of the more powerful, humane C120 Magnum [16] (Figure 1). Since then, a series of mechanically improved Conibear 120 trap models have also been developed and certified as humane by the Fur Institute of Canada [12].

**Figure 2:**
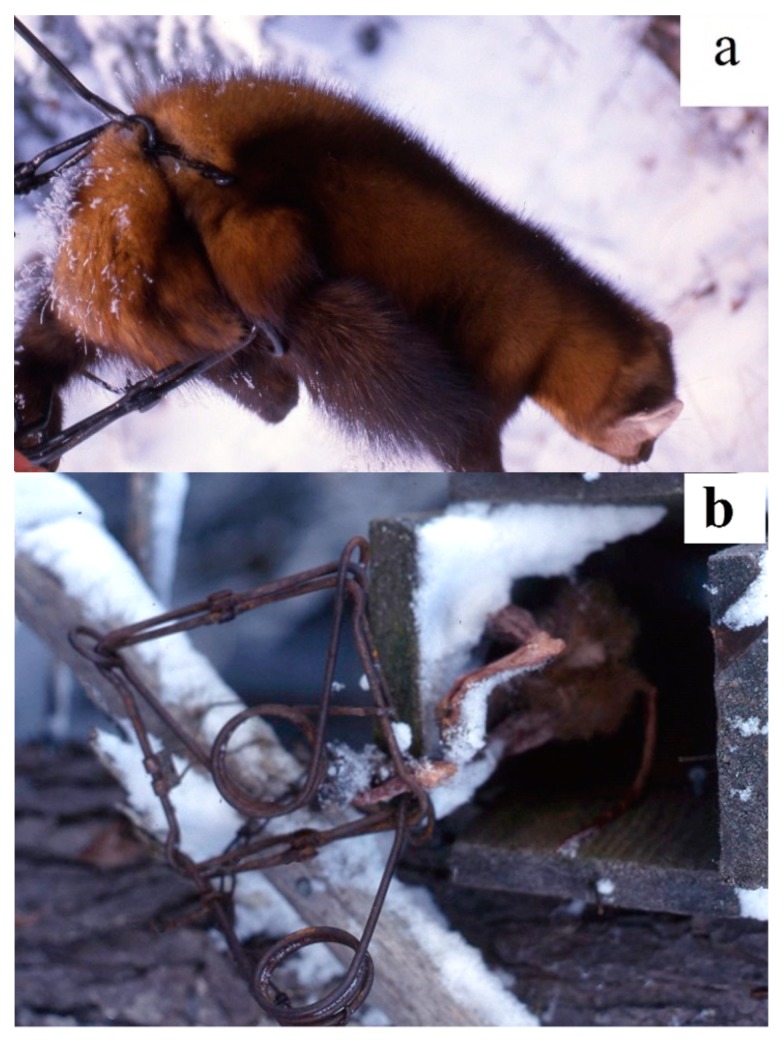
Photographs taken of Conibear 120 captures on working traplines: (**a**) an American marten struck in the lower abdomen; (**b**) this marten was captured by a hind leg and did not succeed in extracting itself from the cubby box where it died. It was later scavenged by other animals as the trapper did not check the trap site in time to retrieve the animal (Photographs: Gilbert Proulx^©^).

All old and new Conibear 120 trap models are sold with a two-prong trigger; the tins of the trigger can be bent and shaped different ways to allow martens to enter the trap, and fire the trigger while attempting to reach a bait placed behind the striking bars. However, martens may bypass the prongs, and go far into the trap before firing the trigger, which results in strikes in non-lethal regions that do not cause an irreversible loss of consciousness in ≤3 min [9] (Figure 2). When animals are captured by the abdomen or legs, they do not die quickly, and killing Conibear 120 traps then become restraining traps. Animals stay alive and commonly die from exposure many hours after capture. Conibear 120 trap models should be equipped with a pitchfork trigger [16] (Figure 1) to ensure that martens are consistently struck in vital regions and die quickly. No matter how powerful Conibear 120 trap models may be, if they are equipped with two-prong triggers, improperly struck martens risk suffering for long periods of time.

In North America, at least 100,000 martens are trapped every year [17,18], but the number of captures may vary from year to year depending on pelt price. The number of martens captured in the Conibear 120 traps is unknown, but due to the popularity of the trap model, it certainly amounts to several thousands of animals. If at least 30% of martens captured in Conibear 120 traps were struck in non-lethal regions [9], then a very large number of martens would likely experience pain and suffering for periods of time exceeding AIHTs’ time limit of 2 min.

### 3.2. The Conibear 120 Trap Model to Kill Mink (Neovison vison)

There are no certified traps for mink in Canada [9] but the Conibear 120 rotating-jaw trap is most popular among trappers. In the USA, the Conibear 120 trap is recommended in BMPs for trapping mink, and neck strikes are identified as proper strike locations [19]. However, as we explained above, the Conibear 120 trap cannot consistently and humanely kill American martens. Mink have a greater cervical musculature and stronger bones than American martens [20], and cannot be humanely killed, i.e., lose consciousness in ≤3 min as per CGSB, by the Conibear 120 trap. In fact, even the mechanically superior and stronger C120 Magnum failed to humanely kill mink captured by the neck [21]. Furthermore, while the Conibear 120 trap is marketed with a two-prong trigger, its inability to properly strike mink in vital regions was reported nearly 50 years ago [22].

The stronger C120 Magnum trap equipped with a pan trigger humanely killed mink double-struck in the neck and thorax [21]. Because the two-prong trigger fails to ensure strikes in vital regions, and the Conibear 120 trap does not have the striking and clamping forces to produce a humane kill, many mink captured in this trap stay alive for many hours, and sometimes until the following day (Rodtka, unpublished data). Thousands of mink are trapped every year in North America [17,18], and many of those captured in the Conibear 120 trap must experience pain and suffering for periods of time exceeding AIHTs’ time limit of 5 min.

### 3.3. Killing Neck Snares for Wild Canids

**Figure 3:**
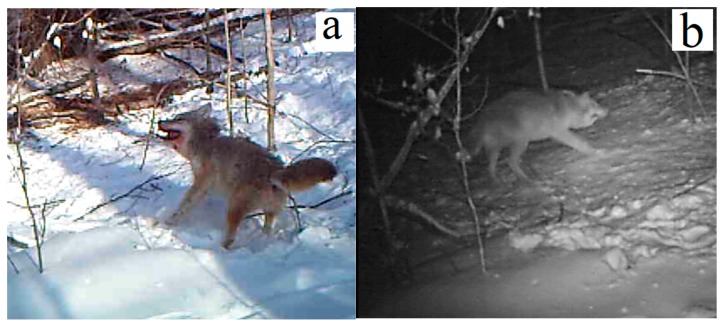
Trail cameras recorded the capture of a coyote in a killing neck snare on a working trapline [10]. The animal was alive for 14 h and 16 min: (**a**) the animal tried to escape at capture time; (**b**) 11 h 30 min later, the coyote was still fighting to escape (Photographs: Gilbert Proulx©).

Killing neck snares are killing devices where the animals, or one or two springs, provide the energy necessary to tighten the noose. These are the most popular kill trapping devices used by trappers because they are cheap, lightweight, easy to set and camouflage, and are efficient at capturing a diversity of furbearers [10]. They are popular in Canada where they are set on traplines to harvest canids, i.e., gray wolves (*Canis lupus*), coyotes (*Canis latrans*) and red foxes (*Vulpes vulpes*) [2,23,24]. Although killing neck snares were originally considered for inclusion in ISO standards [25], which required that snares render captured animals unconscious within 5 min, these trapping devices are not covered under AIHTS [3]. A footnote to Article 7 of the Agreement stipulates that the standards do not prevent individuals from constructing and using traps (which may not pass AIHTS’ time limit test), provided that such traps comply with designs approved by the relevant competent authority. Although killing neck snares are commercially manufactured and sold on the open market [10], they are deemed by competent authority to be non-commercial devices. Certified or not, killing neck snares do not have the ability to quickly and humanely render canids unconscious [26]. Less than 50% of canids captured by the neck in killing neck snares lose consciousness within 300 s [8,27]; death may come after hours or days [28], depending on the killing efficacy of the snare and the frequency of visits by trappers [8,26]. Trail video-cameras set on a working trapline showed that one neck-captured coyote and one wolf lost consciousness after 14 h 16 min and 3 h 39 min of repeated escape attempts, respectively [10] (Figure 3). These videos confirmed years of research showing that killing neck snares do not have the ability to quickly and humanely kill canids [8,26,27]. Although neck snares are sold as devices that are intended to kill, they behave like restraining trapping devices.

More than 100,000 red foxes, coyotes, and wolves are trapped every year in Canada [18], mostly in killing neck snares [10]. Thousands more are snared in the United States [17]. In a previous study of 65 snared coyotes, 59% were neck catches, 20% flank, and 10% foot [29]. Also, nearly half of the animals were alive the morning after being snared. Another study also reported that 5% to 32% of animals captured in various killing neck snare models were still alive when found [28]. While it is best to snare canids behind the jaw where the carotid artery and the trachea are maximally exposed [26], snare location on an animal is influenced by many factors such as the behaviour of the animal when entering the loop [8], snare height and loop diameter, positioning of the lock, preload on the loop (i.e., a little tension is put into the loop to force it to close quicker), and environmental and maintenance factors (rust, twists in the snare cable, snowfall), etc. [26]. Not surprisingly, the percentage of animals found alive in killing neck snares is relatively high [10]. Canids kept alive in killing neck snares die hours or days after being captured, with injuries akin to those recorded with steel-jawed leghold traps [30].

Finally, snared animals may break the snare lock or chew through the cable if the lock does not tighten sufficiently to cause death [28,30]. The likeliness of an escape increases with the length of time an animal is restrained in the killing snare. A 2-year-old male coyote was found in a moribund state on Prince Edward Island, one month after the official end of the trapping season, with a snare deeply embedded in the ventral portion of its neck [31]. Two wolves that had been snared outside Denali National Park and Preserve, Alaska, and had then escaped with the tightened loops around their necks, were spotted by park staff a few days before one of them was immobilized with a tranquilizer dart [32]. The snare was deeply embedded in the wolf’s neck. In both cases, such escapes and injuries could have possibly been avoided with relatively short check time periods [10].

## 4. Stricter Checking Times Are Needed for Killing Traps and Snares

On the basis of past research work, we believe that Conibear 120 traps with two-prong triggers and killing neck snares should be banned altogether [2,26]. However, all killing traps, even those that have been certified as being humane [12], should be monitored frequently because environmental conditions and trappers’ modifications can impact on their killing performance, and one cannot guarantee that all animals will be struck in appropriate locations for a quick kill [2]. Even with certified traps, some animals will not lose consciousness within AIHTS’ time limits and may suffer for long periods of time. When traplines are too long for frequent trap visits, they should be subdivided into smaller sections. Trappers would then be able to check their traps every 24 h, e.g., at sunrise, or even more often. However, when using trapping devices such as killing neck snares that are not considered to be humane by experts who assessed them [26,27,30] but are still being allowed by government agencies, trappers should check them every 12 h. Most carnivores are nocturnal or crepuscular, and the chances to find animals still alive in killing neck snares are greater at dusk and dawn. For example, in Proulx’s video recordings [10], a coyote snared at 11:50 h could have been killed humanely at 17:00 h the same day (trap visit at dusk), instead of 09:00 h the following day (trap visit at dawn, 24 h later).

When traplines are situated near urban areas, checks should be done every 12 h to release pets and non-target animals. In suburban areas, if traps cannot be checked easily, they should be equipped with a monitor [33,34,35,36] that allows false positives but not false negatives, and that notifies a trapper when battery power is low or when a trap has misfired [37].

Our recommendation to frequently check, preferentially every 12 h, killing traps and snares which act like restraining traps is in line with other scientists who recommended that live-holding devices be checked at least daily or more frequently depending upon target species, the potential for capture of nontarget species, and environmental conditions [38]. It is also in agreement with recommendations for the humane and efficient capture of carnivores [2]. Checking traps within a 24-h period on traplines, and within 12 h in urban and sub-urban areas or when using legal but inhumane trapping devices, would minimize pain and discomfort of animals kept alive in killing devices. It would also be advantageous to trappers as it allows them to retrieve captured animals before they are scavenged upon by animals, maintain trap sets that may have been disturbed by animals that avoided capture or by weather conditions, release non-target animals that have not suffered serious injuries during capture, or humanely kill those that are too badly injured to be released.

The concept of humane trapping involves more than just developing devices that meet standards. It also entails changes on how trappers carry out their activities. Shortening trap check times, and using only trapping devices that can consistently and humanely kill animals, would significantly minimize injuries, pain and suffering of trapped animals.

## Appendix A

**Table A1:** Checking times for killing and restraining traps in Canada and the United States of America, 2018–2019.

Jurisdiction	Checking Times			References (2018–2019)	Statement
	Killing Traps and Snares		Restraining Traps		
	Dry Land	Submerged			
**Canadian Provinces and Territories**					
Alberta	No checking time	No checking time	24–48 h	https://open.alberta.ca/dataset/8ccfe254-37d4-42fd-a8ec-fc08fa2fe687/resource/cdd685cf-eaad-4e14-a316-8e8d03da5034/download/albertaguidetrappingregs-2018-2019.pdf	Page 13—Restraining traps: 24 h—Resident Fur Management Licence 48 h—Regional Fur Management Licence
British Columbia	14 days		24–72 h	http://www.env.gov.bc.ca/fw/tmp/hunting-trapping-synopsis-2018-2020.pdf	Page 90—A holder of a licence, permit or other authorization to trap commits an offence unless that person examines the holding or non-killing traps he or she has set on a trapline at least once every 72 h, the egg trap(s) he or she has set for raccoons at least once every 24 h, and killing traps or killing snares that he or she has set on the trapline at least once every 14 days
Manitoba	No checking time	No checking time	72 h	https://www.gov.mb.ca/sd/pubs/fish_wildlife/trapping_guide.pdf	Page 9—No person shall trap fur bearing animals using live holding devices unless they are checked at least once every 72 h.
New Brunswick	No checking time	No checking time	48 h	https://www2.gnb.ca/content/dam/gnb/Departments/nr-rn/pdf/en/Wildlife/HuntTrap.pdf	Page 19—Individual fur harvesters are required to check all restraining trap sets at least once every 48 h. Page 27—Check traps regularly, preferably in the early morning.
Newfoundland and Labrador	No checking time	No checking time	24 h	https://www.gov.nl.ca/hunting-trapping-guide/2019-20/wp-content/uploads/sites/2/Hunting-Trapping-Guide.pdf	Page 96—Fox or Coyote or Lynx Restraining Neck Snare: this is a live capture device and requires a 24 h trap check.
Nova Scotia	No checking time	No checking time	Daily *	https://novascotia.ca/natr/hunt/pdf/Hunting_Summary_2018_Complete.pdf	Page 36—A person who sets cable restraints or traps designed to catch animals alive must examine each trap or snare set at least once every day.
Ontario	No checking time	No checking time	Daily	https://furmanagers.com/wp-content/uploads/2018/08/mnrf-regulations-english.pdf	Page 3—Relaxing cable restraints must be checked on a daily basis.
Prince Edward Island	48 h		Daily	http://www.gov.pe.ca/photos/original/fae_trap02_e.pdf	Page 3—No person shall set a trap designed to hold animals alive without examining each trap at least once a day. No person shall set a trap designed to kill animals without examining each trap at least once every 48 h.
Québec	No checking time	No checking time	No checking time	https://mffp.gouv.qc.ca/english/publications/online/wildlife/trapping-regulations/pdf/trapping-regulation-2018-2020.pdf	
Saskatchewan	1–5 days for killing snares	No checking time	No checking time	https://pubsaskdev.blob.core.windows.net/pubsask-prod/109243/109243-2018_Hunters_and_Trappers_Guide_-_Trapping_Supplement.pdf	Page 2—It is a violation to fail to check traps or snares: within one day when set within five kilometres of urban limits; within one day when setting a mechanically activated leg snare for bears in the SFCA; three days when set on other lands in the southern zones; five days when set on lands within the Fur Conservation Block.
Nunavut	No information found				
Northwest Territories	No checking time	No checking time	No checking time	https://www.justice.gov.nt.ca/en/files/legislation/wildlife/wildlife.r12.pdf	
Yukon	7 days	7 days	5 days	https://yukon.ca/sites/yukon.ca/files/env/env-trapping-regulations-summary.pdf	Every person who installs a snare or trap must: check the set at least once every five days if it is designed to restrain the animal; check the set at least once every seven days if it is designed as a quick killing set.
**United States of America**					
Alabama	24 h	72 h	24 h	https://www.outdooralabama.com/sites/default/files/Hunting/Trapping/Fur%20Catcher%20Code-Regs%201-18.pdf	Section II—K.6: All traps set in or beneath water must be checked at least once every 72 h. All traps other than water sets must be checked at least once every 24 h. Regulation 220-2-.30: Killing neck snares are prohibited.
Alaska	72 h	72 h	72 h	https://www.adfg.alaska.gov/static/regulations/wildliferegulations/pdfs/trapping.pdf	Page 21—All traps/snares must be checked within 3 days of setting them and within each 3 days thereafter.
Arizona	Daily	Daily	Daily	https://repository.asu.edu/attachments/193157/content/2017-18-Trapping-Regulations.pdf	Killing neck snares and body-gripping traps are illegal.
Arkansas	72 h	72 h	Daily	https://agfc.com/en/hunting/furbearers/	Nondrowning sets with foothold traps, snares and box traps must be checked daily. Kill traps must be checked at least every 72 h
California	Daily	Daily	Daily	https://nrm.dfg.ca.gov/FileHandler.ashx?DocumentID=45902&inline	All traps shall be visited at least once daily by the owner of the traps or his/her designee.
Colorado	Daily	Daily	Daily	https://cpw.state.co.us/Documents/RulesRegs/Regulations/Ch03.pdf	Page 2—All traps and snares must be visually checked on site at least once every day.
Connecticut	24 h	24 h	24 h	https://www.somersct.gov/download/Town%20Departments/Town%20Clerk/2019-Hunting-Trapping-Guide.pdf	Page 46—Trappers are required to tend all traps within a 24-h period.
Delaware	24 h	24 h	24 h	http://www.eregulations.com/delaware/hunting/furbearer-trapping-hunting/	It is unlawful to fail to visit traps at least once every 24 h. Only restraining snares are allowed.
Florida	24 h	24 h	24 h	http://www.eregulations.com/florida/hunting/furbearer-regulations/	Live traps and snares must be checked every 24 h. Body-grip traps are prohibited.
Georgia	24 h	24 h	24 h	https://georgiawildlife.com/regulations/trapping	It is unlawful to fail to inspect traps at least once each 24-h period and remove any animals caught in the traps. Killing neck snares are prohibited, except for beaver in water.
Hawaii	No trapping	No trapping	No trapping	https://dlnr.hawaii.gov/dofaw/files/2013/09/HAR-123-Game-Mammals.pdf	Page 28—No person shall possess or use tracer bullets, bullets with full metal jackets, blow guns, guns powered by compressed gas, animal traps, slingshots, poison, explosives, or snares in any public hunting area.
Idaho	72 h	72 h	72 h	https://idfg.idaho.gov/sites/default/files/seasons-rules-upland-furbearer-2018-2019.pdf	Page 36—No person shall place snares or traps for furbearing animals, predatory or unprotected wildlife except pocket gophers, most species of ground squirrels, and other unprotected rodents, without visiting every trap or snare once every 72 h and removing any catch therein.
Illinois	Daily	Daily	Daily	https://www.dnr.illinois.gov/hunting/Documents/HuntTrapDigest.pdf	Page 44—It is unlawful to fail to visit and remove all animals from traps at least once each calendar day.
Indiana	24 h	24 h	24 h	http://www.eregulations.com/wp-content/uploads/2018/07/18INHD_LR7.pdf	Page 43—Traps must be checked and animals removed at least one time every 24 h.
Iowa	24 h	No checking time	24 h	https://bidopportunities.iowa.gov/Home/GetBidOpportunityDocument/91d03035-815c-49c2-afb2-88c30918ab5f	Page 25—All animals or animal carcasses caught in any type of trap or snare, except those that are placed entirely under water and designed to drown the animal immediately, must be removed from the trap or snare by the trap or snare user immediately upon discovery and within 24 h of the time the animal is caught. Mechanically powered snares are prohibited.
Kansas	Daily	Daily	Daily	https://ksoutdoors.com/Hunting/Hunting-Regulations/Furbearers/Trap-Tagging-and-Tending	All traps, including snares and deadfalls, must be tended and inspected at least once every day.
Kentucky	24 h	24 h	24 h	https://fw.ky.gov/Hunt/Pages/Furbearer-Hunting-and-Trapping.aspx	All traps must be visited at least once every twenty-four (24) h and all animals removed.
Louisiana	Daily	Daily	Daily	http://www.wlf.louisiana.gov/sites/default/files/pdf/page/41407-regulations/2018-2019trappingregulations.pdf	All traps must be checked daily.
Maine	3–5 days	1–5 days depending on towns, times of the year, and trap sets.	Unspecified	https://www.maine.gov/ifw/hunting-trapping/trapping-laws/regulations.html#specifictraps	All traps set in organized towns must be tended daily, except for killer-type traps, drowning sets, and under-ice drowning sets. Each killer-type trap or drowning set, except under-ice drowning sets, in organized towns must be tended at least once every three calendar days except if the drowning set is within ½ mile of the built up section of town, then it must be checked every 24 h. All traps set in unorganized towns must be tended daily, except for killer-type traps, drowning sets, and under-ice drowning sets. Each killer-type trap or drowning set, except under-ice drowning sets, in unorganized towns must be tended at least once in every 5 calendar days. During November, December, March and April if a drowning set is under-ice there will be no tending requirement. However, if a trap set is in open water the trap tending requirements are: 3 days for killer-type traps and drowning sets, except if the drowning set is within ½ mile of the built up section of town it must be checked every 24 h, and 5 days for killer-type traps and drowning sets in unorganized towns.
Maryland	Daily	48 h	Daily	http://www.eregulations.com/wp-content/uploads/2018/06/18MDHD_LR.pdf	Page 52—Traps must be checked once per calendar day except those traps that are set in water or tidal marshes which must be checked once per two days.
Massachusetts	Daily	Daily	Daily	https://www.mass.gov/regulations/321-CMR-300-hunting#3-02-5-hunting-and-trapping-of-certain-mammals	It shall be unlawful for any person to fail to visit and remove all animals trapped in, at least once in each calendar day between the hours of 04:00 A.M. and 10:00 P.M., all traps by him staked out, set, used, tended, placed, or maintained.
Michigan	No checking time	No checking time	24–48 h depending on zones	https://www.michigan.gov/documents/dnr/michigan_fur_harvester_digest_625943_7.pdf	Page 24—Trappers are legally required to check traps set in a manner to hold animals alive at least once each day in Zones 2 and 3 and at least once within each 48-h period in Zone 1. It is highly recommended that trappers in Zone 1 check traps daily.
Minnesota	Killing traps: 72 h Snares: daily.	72 h	Daily	https://files.dnr.state.mn.us/rlp/regulations/hunting/full_regs.pdf#view=fit&pagemode=bookmarks	Page 49—Traps capable of capturing a protected animal and not capable of drowning it must be tended at least once each calendar day, except body-gripping traps. Traps capable of drowning the animal and body-gripping traps must be tended at least once each third calendar day, except traps set under the ice.
Mississippi	36 h	36 h	36 h	http://www.mdwfp.com/wildlife-hunting/furbearer-trapping/trapping-regulations.aspx	Every trapper shall visit his traps at least every thirty-six (36) h.
Missouri	Daily	48 h	Daily	https://huntfish.mdc.mo.gov/sites/default/files/downloads/2019HuntTrapRegs.pdf	Page 23—Wildlife must be removed or released from traps daily, except for colony and killing-type traps set under water, which must be checked every 48 h.
Montana	48 h	48 h	48 h	http://fwp.mt.gov/hunting/planahunt/huntingGuides/furbearer/	Page 3—Traps should be checked at least once every 48 h.
Nebraska	48 h	48 h	Daily	http://digital.outdoornebraska.gov/i/1008617-small-game-guide-2018-web	Page 20—Once every two calendar days: Metal spring traps and snares affixed to one-way, slide-wire drowning sets. Underwater snare sets that remain under water when fully extended. Underwater body-gripping sets. Once every calendar day: All others.
Nevada	96 h	96 h	96 h	http://www.ndow.org/Hunt/Seasons_and_Regulations/Furbearer/Trapping_in_NV/	A person taking or causing to be taken wild mammals by means of traps, snares or any other devices which do not, or are not designed to, cause immediate death to the mammals, shall, when the traps, snares or devices are placed or set for the purpose of taking mammals, visit or cause to be visited at least once each 96 h each trap, snare or other device during all of the time the trap, snare or device is placed, set or used in the taking of wild mammals, and remove therefrom any mammals caught therein.
New Hampshire	Daily	72 h	Daily	http://www.eregulations.com/newhampshire/hunting/furbearer-trapping/	A trapper must visit traps set at least once each calendar day. A person trapping beaver through the ice must visit his traps at least once each 72 h.
New Jersey	24 h	24 h	24 h	https://www.njfishandwildlife.com/pdf/2018/trapping_summary18-19.pdf	All traps must be checked and tended at least once every 24 h, preferably in the morning except traps set for semi-aquatic species in tidal waters only must be checked once per calendar day.
New Mexico	Daily	Daily	Daily	http://www.wildlife.state.nm.us/download/publications/rib/2019/hunting/30-Furbearers.pdf	Page 125—A licensed trapper or his/her representative (agent) must personally visit and inspect each trap every calendar day, and all wildlife must be removed. Every other calendar day all traps must be checked personally by the trapper.
New York	24–48 h depending on zones	48 h	24 h		Page 56 In the Southern Zone: You must check traps once in each 24-h period. In the Northern Zone, once in each 48-h period. Traps set in water during the open season for beaver, otter, mink and muskrat, once in each 48-h period. Body-gripping traps set on land, once in each 48-h period. Restraining traps, once in each 24-h period
North Carolina	Daily	72 h	Daily	http://www.eregulations.com/northcarolina/hunting-fishing/trapping-regulations/	Every trap must be visited daily and any animal caught therein removed, except for completely submerged Conibear™-type traps, which must be visited at least once every 72 h and any animal caught therein removed.
North Dakota	No checking time	No checking time	No checking time	https://gf.nd.gov/regulations/small-combined#fur	
Ohio	Daily	Daily	Daily	http://wildlife.ohiodnr.gov/portals/wildlife/pdfs/hunting/2018-19%20Ohio%20Hunting%20Regs_Web.pdf	All traps and snares must be checked and all animals removed once every calendar day.
Oklahoma	24 h	24 h	24 h	http://www.eregulations.com/oklahoma/hunting/furbearer-regulations/	Traps must be tended once each 24-h period.
Oregon	48 h	48 h	48 h	https://www.dfw.state.or.us/resources/hunting/small_game/regulations/docs/Furbearer_Regulations.pdf	Page 2—All traps or snares set or used for the taking of furbearing or unprotected mammals shall be inspected at least every 48 h and all trapped animals removed.
Pennsylvania	36 h	36 h	36 h	http://maps.dcnr.pa.gov/bof/huntmap/pdfs/2018-19%20Hunting%20Trapping%20Digest.pdf	Traps must be visited by the owner once every 36 h, and each animal removed or released.
Rhode Island	24 h	24 h	24 h	http://www.eregulations.com/rhodeisland/hunting/trapping/	All traps must be checked at least once in every 24-h period
South Carolina	24 h	48 h	24 h	http://www.dnr.sc.gov/regs/furharvest.html	All traps must be checked at least once daily from two hours before official sunrise to two hours after official sunset. Body gripping traps used in water sets and other traps used in submersion sets must be checked once every 48 h.
South Dakota	2–3 days	5 days	2–3 days	https://gfp.sd.gov/trapping/	Traps, including snares, must be checked prior to midnight of the second full calendar day (from the time the trap was initially set or last checked) east of the Missouri River and prior to midnight of the third full calendar day west of the Missouri River. Any animal caught must be removed. Traps or snares that are entirely submerged in the water and remain set beneath ice must be checked and any caught animals removed prior to midnight of the fifth full calendar day statewide.
Tennessee	72 h	72 h	36 h	https://www.tn.gov/content/dam/tn/twra/documents/huntguide.pdf	Page 15—Lethal sets such as instant kill traps and water set (“drowning”) traps must be inspected every seventy-two (72) hours. All other traps must be inspected every thirty-six (36) hours and any wildlife caught in the traps shall be removed.
Texas	36 h	36 h	36 h	https://tpwd.texas.gov/regulations/outdoor-annual/hunting/fur-bearing-animal-regulations/means-methods	It is unlawful to take fur-bearing animals with snare, foothold, body grip traps, and live or box trap unless such devices are examined at least once every 36 h and animals are removed.
Utah	96 h	96 h	48 h	https://wildlife.utah.gov/guidebooks/2018-19_furbearer.pdf	Page 15—All trapping devices used to take a furbearer, coyote or raccoon must be checked, and any animals removed, at least once every 48 h. The only exception is if you are using the following types of traps, which must be checked, and have any animals removed, every 96 h: Killing traps that strike the top and bottom of the animal simultaneously Drowning sets Lethal cable devices that are set to capture on the neck, that have a nonrelaxing lock without a stop, and that are anchored to an immovable object.
Vermont	Daily	72 h	Daily	http://www.eregulations.com/vermont/hunting/furbearer-hunting-trapping/	Trappers are required to check their traps at least once a day and dispatch or release any captured animal. The only exception is body gripping traps set in the water or set under the ice, colony/cage traps set underwater, or foothold traps under the ice, which trappers are required to check every three calendar days and remove any animal caught.
Virginia	Daily	72 h	Daily	https://www.dgif.virginia.gov/wp-content/uploads/2018-2019-Virginia-Hunting-and-Trapping-Regulations-Digest.pdf	Page 52—Trappers must visit all traps once each day and remove all animals caught therein, except for completely submerged body-gripping traps which must be visited once every 72 h.
Washington	72 h	72 h	24 h	https://wdfw.wa.gov/sites/default/files/publications/02012/wdfw02012.pdf	Page 2—It is unlawful to trap for wild animals unless traps are checked and animals removed within 72 h (non-body gripping kill traps); and unless animals captured in restraining traps (any nonkilling set) are removed within 24 h of capture.
West Virginia	Daily	Daily	Daily	http://www.wvdnr.gov/hunting/Regs1819/2018-19_Hunting_Regs.pdf	Page 4—All traps must be checked and tended daily.
Wisconsin	Daily	96 h	Daily	https://dnr.wi.gov/files/pdf/pubs/wm/wm0002.pdf	Page 8—non-submersion sets must be attended and checked in person at least once each day; water sets, except submersion sets, must be attended and checked in person at least once each day; submersion sets must be attended and checked in person within a 4-day period following the last tending of the set.
Wyoming	Once per week **	Once per week	72 h	https://wgfd.wyo.gov/Regulations/Regulation-PDFs/REGULATIONS_CH4.pdf	Page 4–8—Check Period for Leg-Hold Traps, Live Traps, Snares and Quick-Kill Body-Grip Traps. (a) All leg-hold traps and live traps shall be checked by the owner a minimum of once during each seventy-two (72) h period. (b) All snares and quick-kill body-grip traps shall be checked by the owner a minimum of one time each week, except during the initial week the snares or quick-kill bodygrip traps were set.

* Daily: the checking period could exceed 24 h if a kill trap/snare was set or checked on a morning of one day and rechecked in the afternoon or evening of the following day. ** Once per week: the checking period could be as long as 13 days if a kill trap/snare was set or checked on a Monday of one week and rechecked on the Sunday of the next week.

## References

[1] Proulx G., Barrett M.W. (1989). Animal welfare concerns and wildlife trapping: Ethics, standards and commitments. Trans. West. Sect. Wildl. Soc..

[2] Proulx G., Cattet M.R.L. (2012). Humane and efficient capture and handling methods for carnivores. Carnivore Ecology and Conservation: A Handbook of Techniques.

[3] ECGCGRF (European Community, Government of Canada, and Government of the Russian Federation) (1997). Agreement on international humane trapping standards. Off. J. Eur. Communities.

[4] (1998). International Agreement in the form of an Agreed Minute between the European Community and the United States of America on humane trapping standards. Off. J. Eur. Communities.

[5] Broom D.M. (1991). Animal welfare: Concepts and measurement. J. Anim. Sci..

[6] Dawkins M.S. (2006). A user’s guide to animal welfare science. Trends Ecol. Evol..

[7] Cook S.R., Barrett M.W., Proulx G. (1989). Assessment and preliminary development of the rotating-jaw Conibear 120 trap to effectively kill marten (*Martes americana*). Can. J. Zoöl..

[8] Proulx G., Barrett M.W. (1990). Assessment of power snares to effectively kill red fox. Wildl. Soc. Bull..

[9] Barrett M.W., Proulx G., Hobson D., Nelson D., Nolan J.W. (1989). Field evaluation of the C120 Magnum trap for marten. Wildl. Soc. Bull..

[10] Proulx G. (2018). Intolerable Cruelty—The Truth Behind Killing Neck Snares and Strychnine.

[11] Fleiss J.L. (1981). Statistical Methods for Rates and Proportions.

[12] Fur Institute of Canada (FIC) Certified Traps—AIHTS Implementation in Canada. https://fur.ca/wp-content/uploads/2015/10/Certified-Traps-List-FIC-April-8-2019-Eng-8½-X-14.docx.pdf.

[13] Association of Fish & Wildlife Agencies (AFWA) (2014). Best Management Practices for trapping American Marten in the United States. https://www.fishwildlife.org/application/files/9115/2105/2578/Marten_BMP_2014_F.pdf.

[14] Canadian General Standards Board (1984). Animal Traps, Humane Mechanically-Powered Trigger-Activated.

[15] Cook S., Proulx G. (1989). Mechanical Evaluation and Performance Improvement of the Rotating Jaw Conibear 120 Trap. J. Test. Eval..

[16] Proulx G., Barrett M.W., Cook S.R. (1989). The C120 Magnum: An effective quick-kill trap for marten. Wildl. Soc. Bull..

[17] Fox C.H., Papouchis C.M. (2004). Cull of the Wild—A Contemporary Analysis of Wildlife Trapping in the United States.

[18] Statistics Canada (2011). Fur Statistics.

[19] Association of Fish & Wildlife Agencies (AFWA) Undated. Best Management Practices for Trapping Mink in the United States. file:///C:/Users/Owner/Documents/Visits%20of%20killing%20traps/MinkRV3.pdf.

[20] Proulx G., Barrett M.W., Cook S.R. (1990). The C120 Magnum with Pan Trigger: A Humane Trap for Mink (*Mustela vison*). J. Wildl. Dis..

[21] Proulx G., Barrett M.W. (1993). Field testing the C120 Magnum trap for mink. Wildl. Soc. Bull..

[22] Cook L., Novak M., Walker L. (1973). Effectiveness of Some Furbearer Animal Traps.

[23] Fédération des Trappeurs Gestionnaires du Québec (FTGQ) (2014). Meilleures Pratiques de Piégeage. http://www.ftgq.qc.ca/fr/publications/images/fiches_piegeage_francais2014.pdf.

[24] Sinnema J. (2014). A country built on fur. Edmont. J..

[25] ISO (1995). Animal (Mammal Traps): Part 4, Non-Mechanically Powered Killing Snares.

[26] Proulx G., Rodtka D., Barrett M.W., Cattet M., Dekker D., Moffatt E., Powell R.A. (2015). Humaneness and selectivity of killing neck snares used to capture canids in Canada: A review. Can. Wildl. Biol. Manag..

[27] Federal Provincial Committee for Humane Trapping (FPCHT) (1981). Report of the Federal Provincial Committee for Humane Trapping.

[28] Phillips R.L. (1996). Evaluation of 3 types of snares for capturing coyotes. Wildl. Soc. Bull..

[29] Guthery F.S., Beasom S.L. (1978). Effectiveness and Selectivity of Neck Snares in Predator Control. J. Wildl. Manag..

[30] Proulx G., Rodtka D. (2017). Steel-jawed leghold traps and killing neck snares: Similar injuries command a change to agreement on international humane trapping standards. J. Appl. Anim. Welf. Sci..

[31] Daoust P.-Y., Nicholson P.H. (2004). Severe Chronic Neck Injury Caused by a Snare in a Coyote, *Canis latrans*. Can. Field-Nat..

[32] Repanshek K. (2008). Vet Removes Snare from Neck of Wolf in Denali National Park and Preserve. http://www.nationalparkstraveler.com/2008/05/vet-removes-snare-neck-Wolf-denali-national-park-and-preserve.

[33] Nolan J.W., Russell R.H., Anderka F. (1984). Transmitters for Monitoring Aldrich Snares Set for Grizzly Bears. J. Wildl. Manag..

[34] Marks C. (1996). A Radiotelemetry System for Monitoring the Treadle Snare in Programmes for Control of Wild Canids. Wildl. Res..

[35] Larkin R.P., Van Deelen T.R., Sabick R.M., Gosselink T.E., Warner R.E. (2003). Electronic signaling for prompt removal of an animal from a trap. Wildl. Soc. Bull..

[36] Néill L.Ó., De Jongh A., Ozolins J., De Jong T., Rochford J. (2007). Minimizing Leg-Hold Trapping Trauma for Otters With Mobile Phone Technology. J. Wildl. Manag..

[37] Powell R.A., Proulx G. (2003). Trapping and marking terrestrial mammals for research: Integrating ethics, standards, techniques, and common sense. Inst. Lab. Anim. Res. J..

[38] Sikes R.S., Gannon W.L., The Animal Care and Use Committee of the American Society of Mammalogists (2011). Guidelines of the American Society of Mammalogists for the use of wild mammals in research. J. Mammal..

